# Effect of water storage on fluoride release and mechanical properties of a polyacid-modified composite resin (compomer)

**DOI:** 10.1016/S1674-8301(11)60034-1

**Published:** 2011-07

**Authors:** Ibrahimm M. Hammouda, Essam E. Al-Wakeel

**Affiliations:** a Dental Biomaterials and; b Department of Dental Biomaterials, Faculty of Dentistry, Mansoura University, Mansoura 35516, Egypt.

**Keywords:** compomer, restorative materials, strength, solubility

## Abstract

We evaluated the effect of water storage on fluoride release and mechanical properties of compomer restorative material. Fluoride release was recorded using a specific fluoride electrode. Flexural properties and fracture toughness were measured using a universal testing machine. Vickers hardness was measured using a micro-hardness tester. There was initial burst of fluoride release up to 1 w, which was diminished to a low level in 1 mon and remained relatively constant over 6 mon. Flexural strength and hardness were increased up to 1 mon followed by a gradual decrease up to 6 mon. Flexural modulus was decreased gradually up to 6 mon. Fracture toughness was increased during the first week and gradually decreased over the storage period. We concluded that flexural properties, fracture toughness, Vickers hardness and fluoride release of compomer were sensitive to water as well as storage time. There was a significant effect of fluoride release on the studied mechanical properties.

## INTRODUCTION

Polyacid-modified composite resin (compomer) restorative materials were introduced as a new class of dental materials to overcome the problems associated with conventional glass ionomers and composite resins[1]. The word compomer is derived from the terms "composite" and "ionomer", indicative that the materials combine the features of both types of these dental materials. Compomer restorative materials are made up of two main constituents: dimethacrylate monomer(s), with two carboxylic groups present in their structure, and filler that is similar to the ion-leachable glass present in glass ionomer cements[2]. Compomers set via light-activated addition polymerization followed by an acid-base reaction that arises from the adsorption of water in the oral cavity[2],[3]. These materials are recommended for cervical lesions, class III and V restorations in adults as well as class I and II restorations in children[3]. As a result of improved physical properties and their ease of handling in comparison to glass ionomer cements, there has been increasing clinical interest in compomers.

Compomers, like composites, do not have the ability to bond to hard tooth structures and require the application of bonding agents[4]–[7]. It was reported that compomers have superior mechanical properties compared with conventional glass ionomers[6]. They release fluoride by a mechanism similar to that of conventional and hybrid glass ionomers. Since compomers have lower amount of glass ionomer material in their formulation, the amount of fluoride and its duration are lower than that of glass and hybrid ionomers[8]–[10].

Compomers take up water and there are significant changes in mechanical properties of these materials when they are placed in aqueous solutions. It has been found that the mechanical properties of compomers are more sensitive to water storage[11]. In the oral cavity, many solutions could potentially affect the behavior of these materials[12]. They are rather sensitive to humidity. Although such materials may behave more like resin composites regarding their properties, water uptake by long-term storage may trigger an acid-base reaction within the material and alter their mechanical properties[13].

The present research was conducted to study the effect of long-term water storage on fluoride release, flexural strength, flexure modulus, fracture toughness and Vickers hardness of compomer restorative material after storage in deionized water for 24 h, 1 w, 1, 2, 3 and 6 mon, and to determine the effect of fluoride release on the studied mechanical properties.

## MATERIALS AND METHODS

The material used in this study was Composan Glass (PROMEDICA, Neuműnster, Germany), and shade A2 was selected. Manipulation of the material was performed according to the manufacturer's instructions. The specimens were polymerized using a halogen light-curing unit (Spring Power, IL, USA) with the curing tip in contact with the glass slide covering the surface of the specimen.

### Fluoride release test

Five disc-shaped specimens were prepared in a split Perspex mold (5 mm in diameter and 2 mm in thickness). The material was packed into the mold and covered on both sides with Mylar strips and microscopic glass slides to extrude the excess material. Polymerization was performed for 40 s using the halogen light-curing unit. After 1 h of curing, each specimen was placed in a plastic container containing 4 mL of deionized water and stored at 37°C. Before each measurement, the specimens were removed from the containers and rinsed with 1 mL deionized water. This water was added to the previous storage water and each specimen was restored in a new container with 4 mL fresh deionized water for further equilibration. Measurements were made for each specimen at an interval of 24 h, 1 w, 1, 2, 3 and 6 mon. Each 4 mL storage water and the 1 mL used for washing were mixed with 4 mL total ionic strength adjustable buffer (TISAB) solution and analyzed for fluoride ions with the use of an ion-specific electrode (Orion Electrode, Orion Research Inc., Boston, MA, USA) connected to an ion analyzer supplied with the measuring unit. The solution was gently stirred during the analysis in a non heated magnetic stirrer. The system was calibrated prior to each evaluation with fluoride standards ranging from 0.1 to 100 ppm. Mean and standard deviation values of fluoride release ions were calculated in ppm for each storage time.

### Flexural strength test

A total of 30 rectangular specimens were prepared, five for each storage time. The specimens were prepared in a custom-made stainless steel mold (25 mm length ×2 mm width ×2 mm thickness). The mold was placed over a glass slab and filled with the Composan Glass paste in one increment. The excess material was extruded using a glass slide. The diameter of the light-curing tip was employed to light-polymerize the specimen over five overlapping areas for 40 s each. Ten min after polymerization, the specimens were stored in deionized water at 37°C. The specimens were aged for an interval of 24 h, 1 w, 1, 2, 3 and 6 mon. A three-point bending test was carried out using a universal testing machine (Type 500, Lloyed Instrument, England) running at a cross-head speed of 0.5 mm/min. A compressive load was applied until fracture of the specimen. Fracture load was recorded and converted to stress through dividing the load by the cross-sectional area of the specimen. Flexural strength (σ) was calculated in MPa according to equation (1)[14]: 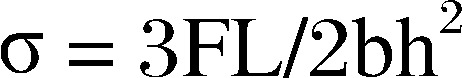
(1)

where F is the failure load in Newton, L is the span length (20 mm), b is the width of the specimen in mm and h is the thickness of the specimen in mm.

During the test, the testing machine recorded load values corresponding to 0.01, 0.03, 0.05 and 0.7 mm vertical displacement. Flexural modulus (E) was calculated in GPa according to equation (2)[14]: 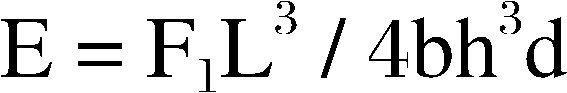
(2)

where F_1_ is the load (N) at a selected displacement point in the elastic region of the stress-deformation plot, L is the span length (mm), b is the width (mm) of the specimen, h is the thickness (mm) and d is the displacement (mm) of the specimen at F_1_. The flexural modulus recorded for each specimen was the average value calculated from the four recorded loads and the corresponding displacements.

### Fracture toughness (K_IC_) test

A total of thirty specimens were prepared, five for each storage period. The specimens were prepared in a custom-made stainless steel mould of (20 mm in length, 4 mm in width and 2 mm in thickness). A sharp blade, forming a part of the mold was used to produce a notch of 2 mm long and a width in the range of 0.45-0.55 mm. A three-point bending test was performed using the Lloyd Testing Machine running at a cross-head speed of 0.5 mm/min until fracture of the specimen occurred. Visual examination of the fractured parts was performed to ensure that the fracture plane was through the notch and perpendicular to the vertical and horizontal planes throughout the center of the specimens. Fracture toughness was determined according to ASTM Designation E 399-83, using equation (3)[15]: 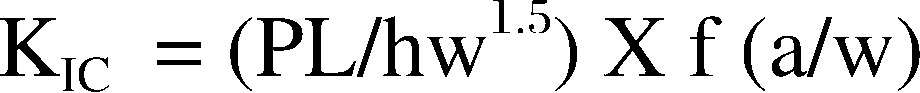
(3)

where K_IC_ is the stress intensity factor, P is the load at fracture, L is the distance between the supports, w is width of the specimen, a is the crack length, and f (a/w) is a function of (a/w). Since the specimen dimensions were based on strict relationship between width, thickness and crack length as imposed by the ASTM standard E399-83, predetermined values of f (a/w) obtained from the standard were used.

### Hardness test

A total of 30 disc-shaped specimens were prepared, 5 mm in diameter and 2 mm in thickness. The specimens were stored and tested at the same conditions and storage times mentioned above. Vickers hardness was measured with a microhardness tester (FM, Future Tech. Corp. Tokyo, Japan). The readings were undertaken using 100 g loading for 15 s. Five indentations were created for each specimen. Mean hardness value was obtained for each specimen. Mean Vickers hardness (kg/mm^2^) and standard deviation were calculated for each storage period.

The results were subjected to one-way analysis of variance (ANOVA) and least significant difference statistical test (LSD) for analysis at *P* = 0.05.

## RESULTS

The mean values of fluoride release are shown in ***Table 1***. One-way analysis of variance revealed that there was a significant difference in the amount of fluoride release among the different storage times (*P* < 0.001). There were a significant decrease in the amount of fluoride release between 24 h and 1, 2, 3, 6 mon of water storage (***Table 1***).

The results of flexural strength and flexural modulus are presented in ***Table 2***. The ANOVA results for both tests showed that there were a significant difference in the flexural strength among the different storage times (*P* = 0.039) and a significant difference in the flexural modulus (*P* < 0.001). In the beginning, there was insignificant increase in flexural strength up to 1 w, followed by a gradual reduction in the subsequent months (*P* = 0.247). On the other hand, the flexural modulus was significantly decreased after 24 h up to 6 mon (*P* < 0.001).

**Table 1:** Mean fluoride release (ppm) and standard deviation values of Composan Glass compomer material at different storage times.

Storage time	Fluoride release (ppm)	SD	*F*	*P*
24 h	2.33^a^	0.30	35.660	0.000
1 w	2.28^a^	0.16		
1 month	1.86^b^	0.21		
2 months	1.73^b^	0.14		
3 months	1.47^c^	0.09		
6 months	1.03^d^	0.15		

Data with different superscripted letters in the same column are significantly different (*P* < 0.05).

**Table 2:** Mean flexural strength, flexural modulus and standard deviation values of Composan Glass compomer material at different storage times.

Storage time	Flexural strength (MPa)	Flexural modulus (GPa)
24 h	127.7±13.7^abcde^	4.0±0.2^a^
1 w	136.0±9.0^bc^	3.9±0.2^a^
1 month	138.0±11.6^c^	2.7±0.2^bc^
2 months	130.2±7.4^cd^	2.7±0.3^c^
3 months	126.9±12.5^de^	2.4±0.3^de^
6 months	114.7±11.0^e^	2.1±0.2^e^
*F*	2.801	52.849
*P*	0.039	0.000

Data with different superscripted letters in the same column are significantly different (*P* < 0.05).

***Table 3*** shows the mean and standard deviation values for fracture toughness and Vickers hardness. One-way ANOVA result for fracture toughness and Vickers hardness showed that there were a significant difference between fracture toughness as well as hardness values among the different storage times (*P* < 0.001). There was a significant increase in fracture toughness in the first week, followed by a gradual decrease over the subsequent months (*P* < 0.001). There was a significant decrease in fracture toughness after 1 w and 6 mon (*P* < 0.001). The results of Vickers hardness fluctuated over the test times. The hardness showed a significant increase after 24 h up to 1 mon (*P* < 0.001), and then a significant decrease up to 6 mon (*P* = 0.033).

The effect of fluoride release on the studied mechanical properties is shown in ***Table 4***. There was a significant effect between fluoride release in the 6 mon and flexural strength, flexural modulus and Vickers hardness (*P* < 0.001). Fluoride release also had a significant effect on fracture toughness of Composan Glass material (*P* = 0.001)

**Table 3:** Mean fracture toughness (K_IC_), Vickers hardness and standard deviation values of Composan Glass compomer material at different storage times.

Storage time	K_IC_ (MPa..m^0.5^)	Vickers hardness (kg/mm^2^)
24 h	1.59 ± 0.10^acd^	29.3±3.8^a^
1 w	2.04 ± 0.14^b^	48.8±4.0^b^
1 mon	1.49 ± 0.12^cd^	64.6±2.4^c^
2 mon	1.49±0.20^d^	58.8±6.5^d^
3 mon	1.23 ± 0.11^e^	38.3±2.0^ef^
6 mon	0.52 ± 0.05^f^	34.9±3.2^f^
*F*	78.014	63.825
*P*	0.000	0.000

Data with different superscripted letters in the same column are significantly different (*P* < 0.05).

**Table 4:** Effect of fluoride release on the studied mechanical properties of Composan Glass compomer material after water storage for 6 mon.

Studied properties	*t*	*P*
Flexural strength	46.660	0.000*
Flexural modulus	5.695	0.000*
Fracture toughness	3.516	0.000*
Hardness	15.833	0.000*

* Highly significant effect of fluoride release.

## DISCUSSION

Modern restorative dentistry is increasingly concerned with the aesthetic outcome of tooth repair as with the restoration of mechanical function and fluoride release. Compomer restorative materials were developed, for such aesthetic repair of teeth damaged by dental caries, with improved mechanical properties as well as ability of releasing fluoride[2],[16]. The results of the present study revealed that Composan Glass material released fluoride during its storage in water. The amount and rate of fluoride release were at its highest level during the first 24 h followed by a rapid decrease up to 6 months[17],[18].

The amount of fluoride released from compomer materials was similar to that released from composites. This implies that, with regard to the fluoride release property, compomers behave more like composites than glass ionomer cements. In addition, the mechanism of fluoride release of compomer occurs only by a diffusion mechanism like composite resin. On the other hand, fluoride release in glass ionomers occurs via both dissolution and diffusion mechanisms[17],[18].

The fact that compomers take up water suggests that there should be significant changes in the mechanical properties of these materials when they are placed in aqueous solutions. The sorbet water not only promotes the acid-base reaction, but also acts as a plasticizer, thereby reducing the strength of the material and possibly altering its failure mode from being predominantly brittle to predominantly tough. Such water sorption may lead to slight swelling of the material[2],[12]. The results obtained from the present study showed that the mechanical properties tested were increased up to 1 w or sometimes 1 mon, then decreased gradually up to 6 mon of water storage. The material showed increase in flexural strength and hardness up to one month and then a gradual decrease up to 6 mon but hardness still higher than that of the 24 h baseline reading. The flexural modulus kept almost constant up to 1 w and gradually decreased up to 6 mon when compared with the 24 h baseline reading. Fracture toughness was increased up to one week, and then gradually decreased up to 6 mon of water storage. The increase in most studied properties in the first week is probably because of increasing the degree of conversion of the double bonds in the resin material present and reducing the amount of free monomer. The decrease in the properties (except hardness) after 1 w indicated dissolution of the material by time. This may be due to PAMCR generally contains more organic matrix and thus may be more susceptible to water absorption and a subsequent surface disintegration in an aqueous environment[19]. These findings indicated that water sorption significantly affects the bulk properties with little effect on surface properties. The results of the present study showed decrease in the mechanical properties, which may be a result of degradation of the investigated material in the aqueous environment[20].

Meyer *et al*.[2] calculated that the diffusion coefficient of water for several polyacid-modified composite resins would take approximately 40-60 d to diffuse 1 mm in linear direction within the materials. The effect of water on the physical properties of PAMCR is controversial. Water may act as a plasticizer, weakening the covalent bonds, degrading components and ultimately decreasing the strength of the material[21]. On the other hand, PAMCR stored in water may undergo a slow-rate, solid-state transformation to produce carboxylate salts that would strengthen the material overtime[22]. The findings of the present study indicated that fluoride release from Composan Glass material had a significant effect on all studied mechanical properties. This was probably because fluoride release from this material occurred by diffusion, which can be either by the release of fluoride in conjunction with an appropriate counter ion, typically sodium, or *via* exchange with hydroxyl groups of the surrounding aqueous environment. Both cases may result in weakening of the material.

In conclusion, within the limits of this study, it was concluded that compomer produced initial burst of fluoride release up to the first week and diminished to a level of release which remained relatively constant over 6 mon. Compomer demonstrated a significantly higher flexural strength up to 1 mon, and gradually decreased up to 6 mon when compared with the baseline (24 h) values after polymerization. Flexural modulus was decreased gradually during storage in water up to 6 mon when compared with the baseline (24 h) value. Fracture toughness of compomer was increased during the first week and gradually reduced over 6 month's storage period. After 6 mon, fracture toughness of compomer reached about one third of the baseline (24 h) value. The hardness was significantly increased up to 1 mon, and gradually decreased up to 6 mon but still higher than the baseline value. The effect of the released fluoride on all mechanical properties was pronounced.
